# Insights into the Distribution Patterns of Foot and Ankle Tumours: Update on the Perspective of a University Tumour Institute

**DOI:** 10.3390/jcm13020350

**Published:** 2024-01-08

**Authors:** Christian Scheele, Andreas Toepfer, Simone Beischl, Dietmar Dammerer, Norbert Harrasser, Rüdiger von Eisenhart-Rothe, Florian Lenze

**Affiliations:** 1 Department of Orthopedics and Sports Orthopedics, Technical University of Munich, Klinikum Rechts der Isar, Ismaninger Str. 22, 81675 Munich, Germany; simone.beischl@mri.tum.de (S.B.); norbert.harrasser@mri.tum.de (N.H.); eisenhart@tum.de (R.v.E.-R.); florian.lenze@mri.tum.de (F.L.); 2 Department of Orthopaedic Surgery and Traumatology, Kantonsspital St. Gallen, Rorschacherstrasse 95, 9007 St. Gallen, Switzerland; andreas.toepfer@kssg.ch; 3 Department of Orthopaedics and Traumatology, Krems University Hospital, 3500 Krems, Austria; dietmar.dammerer@krems.lknoe.at

**Keywords:** foot tumour, musculoskeletal tumour, bone sarcoma, soft tissue sarcoma, distribution pattern

## Abstract

The rarity of foot and ankle tumours, together with the numerous histological entities, presents a challenge in accumulating sufficient patients to draw reliable conclusions. Therefore, we decided to present an update of a retrospective analysis of their distribution patterns, comprising 536 cases of foot and ankle tumours presented to our tumour board between June 1997 and June 2023. Our aim was to provide a comprehensive overview of the prevalence and distribution patterns of benign and malignant bone and soft tissue tumours of the foot and ankle. A total of 277 tumours involved bone (51.7%). Of these, 242 (87.4%) were benign and 35 (12.6%) were malignant. In addition, 259 soft tissue tumours (48.3%) were found, of which 191 (73.7%) were benign and 68 (26.3%) were malignant. The most common benign bone tumours were simple bone cysts, enchondromas, osteochondromas, aneurysmal bone cysts, and lipomas of bone. Common benign soft tissue tumours included a tenosynovial giant cell tumour, haemangioma, plantar fibromatosis, schwannoma, and lipoma. The most common malignant soft tissue tumours were synovial sarcoma, malignant melanoma, and myxofibrosarcoma. In terms of anatomical location, the hindfoot was the most common site (28.7%), followed by the midfoot (25.9%), ankle (25.4%), and forefoot (20.0%). The distribution of benign entities often follows typical patterns, which may facilitate an early diagnosis even without biopsy (e.g., simple bone cyst, plantar fibromatosis). On the other hand, the distribution patterns of many rare or malignant entities are inconsistent. Individual soft tissue malignancies occur very sporadically, even over long periods of time and in specialized tumour centres. It is therefore important to recognise that any suspicious mass in the foot and ankle must be considered a possible malignancy until proven otherwise.

## 1. Introduction

Tumours of the foot and ankle are rare but have significant implications for patients’ mobility and quality of life [1,2,3]. Delayed and incorrect diagnoses are more common than in other regions of the musculoskeletal system as malignancy is often not considered as a differential diagnosis of unspecific lumps and bumps of the foot and ankle [4,5,6]. Understanding the incidence and distribution patterns of these tumours is crucial for an accurate diagnosis, appropriate treatment, and improved patient outcomes [1,7,8,9].

A study by Toepfer et al. examined data from patients treated for tumours of the foot and ankle at our institution between June 1997 and December 2015 [10]. The study found that although the total number of tumours in the foot and ankle region was relatively small compared to the rest of the musculoskeletal system, there was a wide range of potential entities. Given the proportional mass of the foot and ankle, the authors concluded that this region is disproportionately affected by musculoskeletal tumours: the segment weight of a single human foot as the percentage of the total body weight is specified as 1.45 ± 0.126%, including the lateral malleolus [11,12]. The existing literature shows that 5–10% of musculoskeletal tumours involve the foot and ankle though [1,10,13,14]. The rarity of tumours in the foot and ankle, coupled with the variety of possible histological diagnoses, makes it difficult to accumulate enough patients to draw reliable conclusions about specific diagnoses in this anatomic region.

As delayed or inadequate treatment might have a significant impact on both oncologic and functional outcomes, we decided to update the work of Toepfer et al. with all patients subsequently treated at our institution in the period from January 2016 to June 2023 [15]. The aim of our retrospective epidemiological study was to provide a comprehensive overview of the prevalence of bone and soft tissue tumours of the foot and ankle, their entities, and their anatomical distribution, covering more than a quarter of a century of patient data and 536 cases.

## 2. Materials and Methods

In this study, we identified all patients with foot and ankle tumours who were discussed in our multidisciplinary tumour board between January 2016 and June 2023. The inclusion criteria consisted of presentation of tumours involving the foot and ankle, a histologically confirmed diagnosis, and treatment at our institution. Patients were excluded if there were insufficient data, including missing medical records, imaging studies, or histological verification of the diagnosis, which hindered accurate identification of the tumour. Foot and ankle tumours were classified based on the WHO classification of musculoskeletal tumours, encompassing neoplastic tumours while excluding tumour-like lesions and pseudotumours, such as ganglion cysts, Morton’s neuroma, and bursitis. Foot and ankle tumours originating from other organs such as skin tumours (e.g., malignant melanoma, poroma) and metastases were also included in our study as treatment was performed by orthopaedic surgeons (tumour orthopaedic and foot and ankle surgeons), following the diagnostic and therapeutic principles of musculoskeletal oncology. The relevant WHO classifications were also used to categorise these tumours.

The talo-crural articulation represents an inherent functional part of the human foot. Therefore, Toepfer et al. proposed a modified anatomical classification of the foot skeleton, including the distal tibia and fibula as a separate localization in their study [10]. Similar to Ruggieri’s classification [13], this classification distinguishes between the forefoot (phalanges), midfoot (metatarsals and lesser tarsals), and hindfoot (talus and calcaneus), but adds the ankle (distal tibia and fibula). The ankle was not explicitly mentioned as a distinct anatomical region by Ruggieri et al. and includes the epi-metaphysis of the distal tibia and fibula, as determined by the AO classification (a square equal in length to the widest part of the growth plate of the tibia and fibula). Where a soft tissue mass extended across multiple anatomical compartments, the presumed centre of the lesion was assigned to the corresponding underlying bone or anatomical region.

Patient information, including age at treatment, sex, side, histological confirmation of the diagnosis, and anatomical location, was collected. A comprehensive evaluation of all available imaging studies such as plain radiographs, magnetic resonance imaging, and computed tomography was performed. The histological classification of each tumour was verified by a certified musculoskeletal pathologist. The variables analysed in this study included tissue origin (bone or soft tissue), categorization as benign or malignant, anatomical location (forefoot, midfoot, hindfoot, or ankle), and specific histological subtype.

The data collection and analysis were performed utilizing Microsoft Excel software (Microsoft Excel version 16.78, Microsoft, Richmond, WA, USA). Categorical variables were reported as frequency counts and percentages of the total number of lesions in each category. A descriptive statistical analysis was conducted for demographic data, including mean values, standard deviations, and minimum/maximum values where applicable.

## 3. Results

### 3.1. Analysis of the Additional Period (2016–2023)

During the period between January 2016 and June 2023, a total of 3154 bone and soft tissue tumours were discussed in our multidisciplinary tumour board. Among these, 223 cases were identified as foot and ankle tumours in 220 patients, of which only 123 cases in 122 patients met the inclusion criteria. The excluded cases consisted of pseudotumours, tumour-like lesions, or nonspecific histologic findings.

There were 62 tumours found on the right side, and 61 on the left side. The study involved 55 (44.7%) male patients and 68 (55.3%) female patients. The gender distribution among patients with benign tumours was 43 males to 55 females, while for all malignant tumours, it was 12 males to 13 females.

Out of the 123 analysed foot and ankle tumours, 98 (79.7%) were classified as benign and 25 (20.3%) were classified as malignant. Furthermore, 11 (8.9%) were bone tumours and 112 (91.1%) were soft tissue tumours. While no malignant entities were observed among the 11 osseous lesions, the 112 soft tissue lesions were divided into 87 benign and 25 malignant cases. Of all the cases observed, 18.7% occurred on the forefoot, 43.1% on the midfoot, 23.6% on the hindfoot, and 14.6% on the ankle (Table 1).

The average age of all patients at the time of diagnosis was 45.2 ± 17.7 years, ranging from 7 to 92 years. The mean age of all patients with osseus tumours was 39.4 ± 15.0 years, ranging from 7 to 67 years, and for all soft tissue tumours, it was 45.7 ± 17.8 years, ranging from 10 to 92 years. For benign soft tissue tumours, the average age was 42.3 ± 16.2 years, ranging from 10 to 90 years, while for malignant soft tissue tumours, the average age was 57.6 ± 18.0 years, ranging from 13 to 92 years.

**Figure 1:**
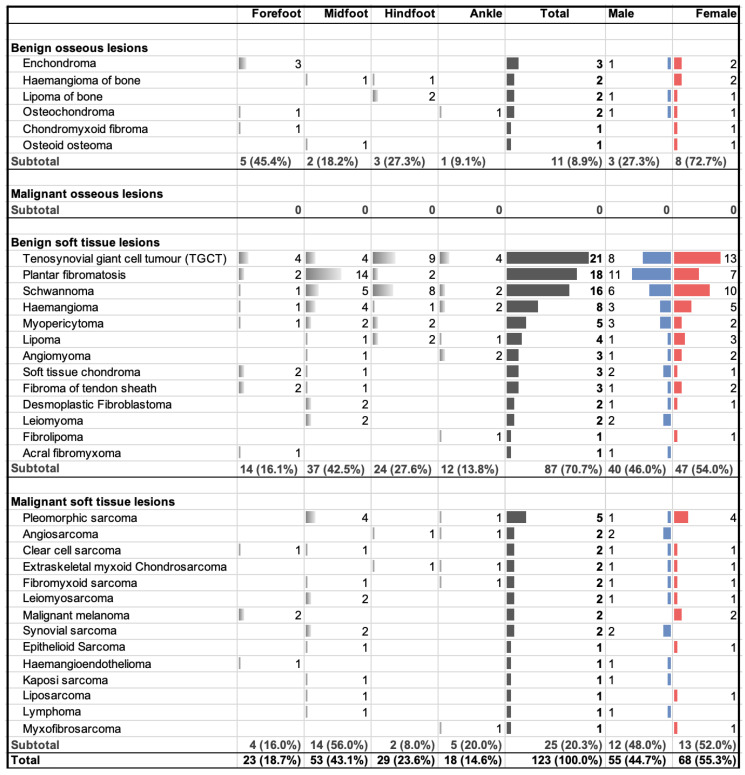
2016–2023: Distribution of benign and malignant osseous and soft tissue tumours across anatomic location and gender.

A total of 33 different entities were identified, including 14 malignant and 19 benign entities, excluding pseudotumours and tumour-like lesions (epidermoid cysts, ganglion cyst, intraosseous ganglion, Morton’s neuroma). There were 6 different entities among the 11 bone tumours. The most common was enchondroma, which accounted for three cases. Most of the entities appeared at the forefoot (45.4%). Two of three patients were female (Figure 1). Between 2016 and June 2023, no malignant bone tumours at the foot and ankle were presented in our multidisciplinary tumour board.

Benign soft tissue tumours accounted for 70.7% of all tumours in the additional study period. A variety of 13 different entities were observed among those 87 benign soft tissue tumours. A tenosynovial giant cell tumour (TGCT) was the most prevalent entity in this category, accounting for 21 cases (24.1%), followed by plantar fibromatosis with 18 cases (20.7%), schwannoma with 16 cases (18.4%), and haemangioma with 8 cases (9.2%). These four entities represented 72.4% of all benign soft tissue tumours. Overall, the midfoot and hindfoot were most frequently affected, representing 61 of the 87 manifestations.

Out of the additional 123 tumours, 25 cases (20.3%) were malignant soft tissue tumours, representing 14 different entities. The most prevalent type among these malignant tumours was undifferentiated pleomorphic sarcoma with five cases (20.0%). Angiosarcoma, clear cell sarcoma, extraskeletal (myxoid) chondrosarcoma, fibromyxoid sarcoma, leiomyosarcoma, synovial sarcoma, and malignant melanoma were found in two cases each. All other entities in this category occurred only once. Malignant soft tissue tumours were most commonly situated at the midfoot (56%), followed by the ankle (20%), the forefoot (16%), and the hindfoot (8%).

### 3.2. Analysis of the Total Study Period (1997–2023)

**Figure 2:**
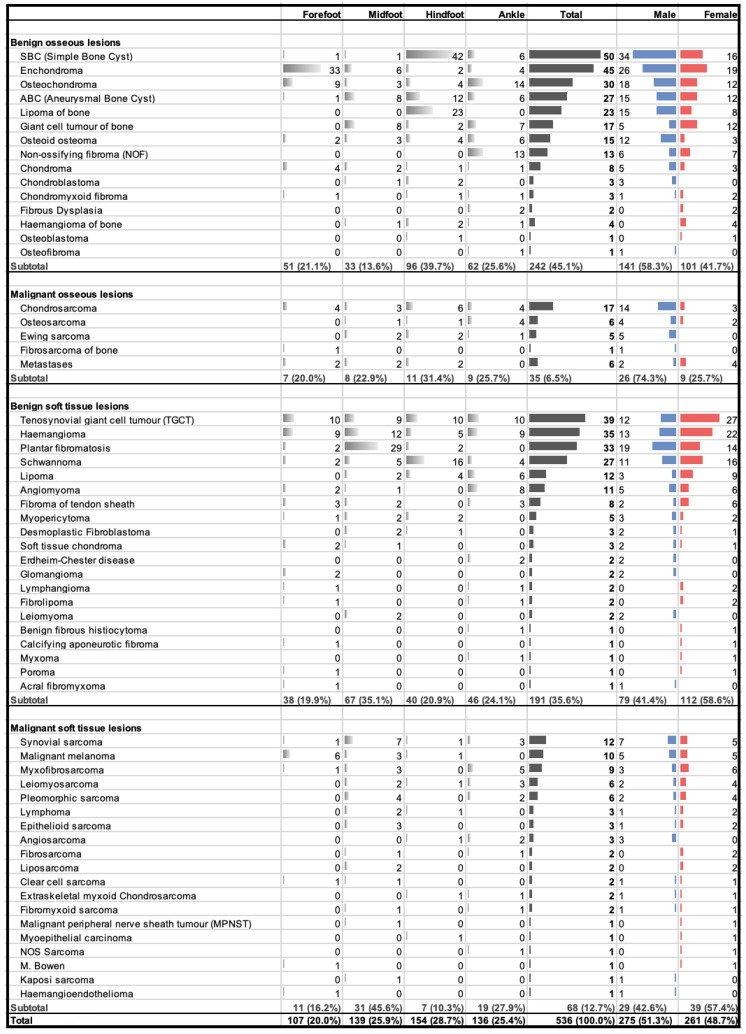
1997–2023: Distribution of benign and malignant osseous and soft tissue tumours across anatomic location and gender.

During the total study period from June 1997 to June 2023, 536 cases met the inclusion criteria (Figure 2). This represents the combined results of the initial study by Toepfer et al. (1997–2015) [10] and the above-mentioned additional period (2016–2023).

Due to the limited number of newly added benign bone tumours, the numbers are almost unchanged from the previous study and the composition of the five most common entities stayed the same. Furthermore, our findings showed no cases of malignant bone tumours in the foot and ankle during the added period, and therefore, the frequency data for malignant bone tumours correspond to the original study period from 1997 to 2015.

The number of soft tissue tumours increased significantly by 112, from 147 to 259. As a result, the percentage of soft tissue tumours increased from 35.6% to 48.3% of all foot and ankle tumours in this study. In the soft tissue subgroup, the number of benign entities increased from 104 to 191 and the number of malignant entities increased from 43 to 68. Concerning the top five most common diagnoses of benign soft tissue tumours, TGCT has become the most common diagnosis overall (*n* = 39), surpassing haemangioma (*n* = 35). Contrary to the first study, TGCT was increasingly recorded in the hindfoot region, leading to a balanced distribution. Furthermore, the four entities of soft tissue chondroma, leiomyoma, acral fibromyxoma, and myopericytoma have been newly diagnosed, expanding the benign soft tissue tumour group to 20 different entities. A total of 5 new entities have been added to the group of malignant soft tissue tumours between 2016 and 2023, representing a rise of +35.7% (from 14 to 19 different entities). Undifferentiated pleomorphic sarcoma has now taken the place of fibrosarcoma as the fifth most common entity, and malignant melanoma has overtaken myxofibrosarcoma in the top five.

The prolongation of the study period has marginally increased the proportion of tumorous lesions in the midfoot from 21.5% to 25.9%. The overall sex and side ratio has levelled off. The ratio of soft tissue tumours to bone tumours was also affected in the analysis since significantly more soft tissue tumours were added. Furthermore, the overall malignancy rate increased from 18.9% to 19.2% but decreased in both subgroups, from 13.2% to 12.6% for osseus tumours and from 29.3% to 26.3% for the soft tissue tumours.

## 4. Discussion

Foot and ankle tumours are rare entities, accounting for approximately 5–10% of all musculoskeletal tumours [1,10,13,14]. In our analysis, they represent 536 of 10,641 bone and soft tissue tumours discussed in our multidisciplinary tumour board between June 1997 and June 2023. This finding is consistent with previous studies, most notably with the underlying publication by Toepfer et al., who reported a percentage of foot and ankle tumours of 5.5%, Chou et al., who reported 5.8% of 2660 tumours, and Ozdemir et al., who reported 10.9% of 1786 tumours. In a study of 39,179 soft tissue tumours, Kransdorf et al. found that 5% of all malignant lesions and 8% of all benign lesions were in the foot and ankle [1,10,13,14,16,17,18]. Understanding the incidence and distribution patterns of foot and ankle tumours is critical for correct diagnoses and treatment [19].

The average age of patients in the additional period was 45.2 years at the time of diagnosis, almost 10 years higher than in the initial period from 1997 to 2015 evaluated by Toepfer et al. (36 ± 18) and 2 years higher than in a study by Ruggieri et al. [10,13]. Of all 123 added tumours, 50.8% were located on the right, and 49.2% on the left side. A total of 44.7% affected male patients and 55.3% affected female patients. Over the whole study period, 51.3% of cases involved men and 48.7% involved women. In men, bone tumours (58.3% of benign tumours and 74.3% of malignant tumours) were more common, whereas soft tissue tumours occurred more frequently in women (58.6% of benign tumours and 57.4% of malignant tumours).

In the additional period between January 2016 and June 2023, the overall malignancy rate was 20.3%, which can be attributed to the rate of malignancies in soft tissue tumours, which was 22.3%. No osseous malignancies were diagnosed in that period. This supports the lower malignancy rate of bone tumours compared to soft tissue tumours in the previous period of 13.2% vs. 29.3% [10]. The lower malignancy rate within soft tissue tumours between 2016 and 2023 compared to the period between 1997 and 2015 may be the result of increased awareness, resulting in even more unclear and ultimately benign tumours being presented to the tumour board. This would also be consistent with the greater proportion of soft tissue tumours among the added 123 tumours of 91.1% compared with 35.6% in the initial study. Due to the higher proportion of soft tissue tumours, the overall malignancy rate increases slightly from 18.9% to 19.2%, while it decreases in both subgroups from 13.2% to 12.6% for bone lesions and from 29.3% to 26.3% for soft tissue lesions. In a study by Ruggieri et al., malignancy rates were higher with rates of 20.6% for bone tumours, 51.8% for soft tissue tumours, and 25.6% for the total cohort [13]. Pollandt et al. (2003) reported a rate of 20.4% for malignancy in their series of bone tumours of the foot and ankle [17]. Differences in malignancy rates between publications may be due to national healthcare structures. In some countries, the treatment of malignant tumours of the musculoskeletal system is reserved for a very small number of centres, while in other countries, the caseload is shared between multiple institutions. In addition, the lower overall malignancy rate in our study and the lower malignancy rates in both subgroups may reflect the aforementioned increased awareness, leading to a lower threshold for presentation of unclear masses to our multidisciplinary tumour board. However, the rates are consistent with previous literature, which, although varying slightly from study to study, generally reports that malignant tumours account for less than 25% of all foot and ankle tumours [1,13,20].

While metastases are by far the most common malignant tumours of the musculoskeletal system in general, their prevalence in the foot and ankle is negligible, accounting for less than 1% and typically occurring late in the course of disseminated disease, resulting in a poor prognosis [21,22,23]. It is therefore not surprising that no cases of metastases were presented to our multidisciplinary tumour board during the entire period. According to a recent literature review, the most common primary tumour appeared to be lung cancer, followed by endometrial and breast cancer [24].

The incidence of bone tumours was found to be 8.9%, which is significantly lower than the 64.4% reported in the previous study period. Considering the small absolute number of only 11 cases since 2016, and considering the previously reported malignancy rate of 13.2%, it is not surprising that there were no malignant bone tumours in the additional period [10]. However, also due to the limited number of cases, there were no significant changes in the findings regarding bone tumours. No new entities were identified during this period, and simple bone cysts (SBCs), enchondroma, and osteochondroma remain the most frequently diagnosed bone lesions. The reasons why so few osseous lesions have been observed in recent years remain unclear.

The overall preference for benign bone tumours in the hindfoot and ankle, observed in the original study period, cannot be confirmed in the subsequent study period from 2016 to 2023. It should be noted, however, that it is necessary to look at the distribution patterns at the level of the entities. Entities that occurred most frequently or exclusively in the ankle, such as osteochondroma or non-ossifying fibroma (NOF), were not added in significant numbers. The only two added cases of osteochondroma occurred in the forefoot and ankle, which is in line with the distribution patterns described by Toepfer et al., with 46% of osteochondromas occurring at the ankle and 29% at the forefoot [10]. This is also true for lesions with a preference for the hindfoot, like SBC, lipoma of bone, or aneurysmal bone cyst. The two new cases of IOL were both located in their pathognomonic location, Diard’s area 6, between the major trabecular groups of the calcaneus, where Toepfer et al. described all lipoma of bone in their series [10,25]. Interestingly, several studies have demonstrated that IOL can develop from SBC and might be considered a developmental stage of SBC, often times showing cystic and lipomatous changes next to each other on preoperative MRI, intraoperative endoscopic visualization, and a postoperative histopathologic analysis [26,27,28]. This explains why only the age of the patient and not the location within the foot skeleton differs between SBC and lipoma of bone. No entity showed a strong preference for the midfoot, although a giant cell tumour, an aneurysmal bone cyst, or enchondroma can regularly be found there. In our study, the latter demonstrates a preference for the forefoot, with 73.3% of lesions occurring in the phalanges. This is consistent with the updated findings, as all three additional cases of enchondromas were localized in the forefoot. Enchondroma is known to be a relatively common entity, accounting for about 10% of all benign bone tumours, with about half occurring in small bones such as those in the hands or feet [29].

Among malignant bone tumours, chondrosarcoma was the most common entity during the original study period, accounting for nearly half of the 35 cases recorded. More than 50% of these cases were located in the ankle and hindfoot areas [10]. Although chondrosarcoma of the foot represents only 1% to 3% of all chondrosarcomas, they are recognized as one of the most common malignant bone tumours of the foot and ankle [30,31]. Most cases are primary tumours, with only a minority arising from pre-existing benign bone lesions such as Ollier’s disease [32,33,34,35,36,37]. A study by Dahin and Unii found that out of 774 primary chondrosarcomas, only 14 (1.8%) were located in the foot, while secondary chondrosarcomas were not observed in this area [30]. However, cases of chondrosarcomas arising from enchondromas have been reported, although such cases are rare [33,35,36,38]. According to Ruggieri et al., chondrosarcoma was found to be the second most common malignant bone lesion in the foot with 14.4% of all bony malignancies following Ewing’s sarcoma with 21.8% [13]. They primarily affect individuals in their 40s to 50s and are more commonly found in the hindfoot, with approximately half of the cases involving the calcaneus [2,39,40].

Osteosarcoma is a malignant tumour that develops relatively rarely in the foot, accounting for only 1% of all cases. It is slightly less uncommon at the distal tibia or fibula, occurring in about 2.5% of cases, which is in line with our results, which show that two thirds were registered at the ankle and one third in the rest of the foot. In contrast to osteosarcoma of the most typical sites (distal femur, proximal tibia, proximal humerus), osteosarcoma of the foot usually affects adults from the fourth decade of life [15,41,42]. Ewing’s sarcoma, although uncommon in the foot and particularly rare in the small bones of the feet, is still considered the most common malignancy affecting foot bones in children. The talus, calcaneus, and metatarsals are frequently affected sites for this type of tumour. The time to diagnosis, also known as the diagnostic delay, is particularly high for both entities when localised in the foot and ankle. In 1997, Adkins et al. showed that highly malignant Ewing’s sarcoma of the foot and ankle was not correctly diagnosed until an average of 14 months after the onset of symptoms, 7 months in the forefoot and 22 months in the hindfoot [43]. Unfortunately, little has changed since then: in a 2013 study by Brotzmann et al., the diagnostic interval for foot tumours was 64.5 weeks for osteosarcoma and 77.4 weeks for Ewing’s sarcoma [5].

The number of soft tissue tumours increased significantly from 147 to 259 through the extension of the study period. Consequently, the proportion of soft tissue tumours rose from 35.6% to 48.3%. Within the soft tissue subgroup, there was an increase in the number of benign entities from 104 to 191, as well as an increase in the number of malignant entities from 43 to 68.

There was no change in the composition of the five most common benign soft tissue entities. TGCT (*n* = 39), haemangioma (*n* = 35), plantar fibromatosis (*n* = 33), schwannoma (*n* = 27), and lipoma (*n* = 12) make up 76.4% of benign soft tissue tumours. TGCT, as the most common entity overall, and haemangioma were evenly distributed among the different regions. Plantar fibromatosis showed a predilection for the midfoot and schwannoma for the hindfoot, and lipoma was less common in the distal areas.

TGCT was the most common benign soft tissue lesion with 39 cases. This rare synovial lesion affects joints, tendon sheaths, and bursae, and is reported as one of the most common soft tissue tumours in the foot and ankle [10,14,44]. With 14% of diffuse TGCTs, the ankle is the second most affected joint after the knee with 64% [45]. Nonetheless, TGCT may develop anywhere in the foot and ankle region, which is consistent with our results [46]. In our study, 27 female and 12 male patients were observed to have TGCT. Previous research suggests that roughly 55% of all cases involve female patients, and onset typically occurs in the third to fifth decade of life [46,47,48]. Whenever feasible, surgical excision remains the preferred treatment option.

Haemangioma was the second most common benign soft tissue tumour in our study. According to a study conducted by Kransdorf et al. (1995), it was found in 8% of 38,484 individuals and 14% of these lesions occurred in the lower extremity [16]. Various studies of lower extremity haemangiomas have reported incidences ranging from 4.9% to 28.5% within the foot [49,50]. In our analysis, haemangioma showed a tendency to occur distal to the Chopart’s joint and affected more females (*n* = 22) than males (*n* = 13).

Plantar fibromatosis, also known as Ledderhose disease, was found in 33 cases and is a benign fibroblastic tumour that originates in the plantar fascia, usually in the mid to distal aponeurosis, and presents as a mass at the plantar aponeurosis on MR imaging [3,51]. Our results are consistent with previous reports that plantar fibromatosis is more commonly observed in males [52]. However, it was reported that plantar fibromatosis is the most common soft tissue lesion in the foot [53], which was not reflected in our analysis. An explanation for this might be that thickening and proliferation of the plantar fascia is a typical finding that is often treated outside of established tumour centres.

The fourth most common diagnosis of a benign soft tissue tumour was schwannoma, which was observed in 27 cases. Schwannomas are slow-growing solid tumours that develop from Schwann cells in the peripheral nerves [54,55]. They are relatively infrequent in the foot and ankle region, with only 9.2–11.5% of schwannomas being observed in that area [13,14,55,56,57]. Ruggieri et al. (2014) reported 14 instances of schwannomas in the foot out of 45 benign soft tissue tumours observed [13]. Our analysis found that schwannomas were more likely to occur in the hindfoot with 59.3% of all cases observed in this area. Schwannomas, however, can occur in any part of the foot. While our observations showed a higher incidence of schwannomas among females (*n* = 16) compared to men (*n* = 11), Hao et al. found no gender differences [54].

Regarding soft tissue malignancies, our latest analysis adds 25 cases distributed across 14 different entities to the 43 cases of the previous study. Five of these fourteen entities represent entities not observed in the previous study. This illustrates that malignant soft tissue tumours of the foot are a very heterogeneous group, with individual entities occurring only very sporadically, even over long periods of time and in specialized tumour centres.

Overall, synovial sarcoma remains the most common soft tissue malignancy of the foot with 12 out of 68 cases. This is in line with previous studies, which observed synovial sarcoma as the most frequent malignant soft tissue tumour of the foot and ankle [13,16,56,58]. Although its name may be misleading, this tumour is typically found close to joints and rarely originates from a synovial space [59,60]. WHO’s classification of bone and soft tissue tumours lists synovial sarcoma within the group of tumours of uncertain differentiation and as a monomorphic blue spindle cell sarcoma showing variable epithelial differentiation. The growth is typically slow, and patients may initially experience a painless swelling, which can easily be misdiagnosed as a cyst or plantar fibromatosis [51,59,61].

Epithelioid sarcoma and clear cell sarcoma, both very rare types of soft tissue tumours, have a strong predilection for the foot and ankle and are reported to affect the foot and ankle more frequently than other sites [51].

The second most common malignant entity in our study is malignant melanoma, although it is a skin malignancy rather than a musculoskeletal one. About half of the melanomas located in the hand and foot are of the specific acral lentiginous melanoma subtype, which occurs primarily in the palms, soles, and nail beds [62]. In the added period, both cases involved toes and resulted in amputation. Unfortunately, patients with malignant melanoma of the foot and ankle have significantly lower 5- and 10-year survival rates compared to melanoma at other sites [63].

Although the compact anatomy should facilitate early detection of tumours of the foot and ankle, a timely diagnosis is often missed based on a lack of awareness. In general, the surgical management of foot and ankle tumours does not differ from that of other localizations and is based on the established principles of classification and treatment of benign and malignant musculoskeletal neoplasms according to Enneking [42]. Benign inactive or latent tumours (Enneking Grade 0, stage 1) do not always require therapy and can warrant follow-up controls or no action at all. These so-called “do not touch” or “leave me alone” lesions are so characteristic radiographically, that further diagnostic tests such as a biopsy are unnecessary and can be frankly misleading and lead to additional unnecessary surgery. A typical example of an osseous “do not touch”-lesion is the NOF. Other entities that usually do not require biopsy include SBC or IOL of the calcaneus, as imaging with plain radiographs and MRI is so typical that histopathological confirmation of the diagnosis is usually not required [64]. Still, critical size (Pogoda criteria), pain, or tumour anxiety can warrant surgery [28,65] for SBC or IOL [28,65]. If a suspicious lump or bump of the foot and ankle cannot be further distinguished using imaging diagnostics, a histopathological analysis through open or image-guided biopsy must be pursued [9,42].

Surgery remains the mainstay of treatment for malignant tumours. Wide resection is required for all intermediate to high-grade tumours (≥G2) in a curative setting. The need for (neo)adjuvant therapy must be discussed on an individual basis in a specific musculoskeletal tumour board. No oncologic compromises should be made when planning tumour resection for intermediate or poorly differentiated sarcomas. Unfortunately, both wide and radical resection are often tantamount to amputation due to the anatomical limitations of the foot. These findings accentuate the importance of an early diagnosis and initiation of therapy, to which the present study aims to contribute [42].

This is the second largest study on the distribution patterns of foot and ankle tumours to date, following the study by Ruggieri et al. [13]. However, our study has several limitations that may affect our conclusions. First, all data were collected from a single centre, which may limit the applicability of our findings. In addition, the cases referred to our centre for musculoskeletal tumour patients may represent more advanced or symptomatic cases, introducing a potential selection bias. Another limitation is that most cases in our review involved surgery, which may have excluded benign and asymptomatic cases that were not discussed in our multidisciplinary musculoskeletal tumour board. In addition, the predominant inclusion of Caucasian patients in our study may limit the generalizability of our findings to patients of different ethnicities. Moreover, it is important to recognize that there is an alternative classification system for anatomic regions of soft tissue tumours and tumour-like lesions of the foot. Kirby et al. proposed a classification that divides the foot into five zones: the ankle, heel, dorsum of the foot, plantar surface of the foot, and toes [58]. Although this classification has been used by other authors [8,66], we chose to use the anatomical classification proposed by Ruggieri et al. to allow a direct comparison with their data and a previous study. Kirby’s classification is not applicable to osseous lesions, so a rigorous analysis and comparison of osseous and soft tissue lesions is not possible [10]. Regarding statistical and data limitations, it should be noted that certain entities are extremely rare, even over an extended period, which was more than a quarter century in this updated study. As previously noted by Chou et al. (2009), the low incidence of foot and ankle tumours, coupled with the wide range of possible histologic diagnoses, makes it difficult to accumulate a sufficient number of patients to draw reliable conclusions about specific diagnoses in this anatomic region [14].

## 5. Conclusions

True foot and ankle tumours are less common compared to non-neoplastic pseudotumour lesions, and it is crucial for physicians to consider the possibility of aggressive or malignant neoplastic disease when evaluating patients with unclear foot lesions. A timely diagnosis and appropriate treatment play an essential role in improving the prognosis and functional outcomes for these patients.

The anatomical distribution of various benign entities often follows typical patterns that may facilitate an early diagnosis. However, the location of various rare or malignant entities lacks consistency. Therefore, while our findings provide helpful information alongside existing data, they cannot serve as a definitive map of where to find a specific tumour entity. It remains crucial to recognize that any suspicious mass of the foot and ankle should be regarded as a potential malignancy unless proven otherwise. In the interest of the patient and due to the complexity of this heterogeneous pathology, the expertise of a musculoskeletal tumour centre including a tumour–orthopaedic surgeon or tumour-trained foot and ankle surgeon should be consulted in unclear cases.

The findings of this study on the distribution patterns and different tumour types may improve our understanding of the diverse pathology of foot and ankle tumours, potentially allowing for better therapeutic outcomes. The results also highlight the marked heterogeneity in the diagnosis of foot tumours, emphasizing the importance of a careful clinical analysis and evaluation of ankle and foot tumours. Further research and data collection is needed to gain a better understanding of specific diagnoses, treatment strategies, and long-term outcomes related to foot and ankle tumours. The extent to which emerging technologies such as artificial intelligence or a big data analysis will aid in clinical diagnostics in the future is yet to be determined and should be the focus of future scientific research.

## Figures and Tables

**Table 1:** Distribution of osseous and soft tissue tumours according to localization and dignity.

Location	Bone	Soft Tissue	Total
Forefoot	5 (21.7%)	18 (78.3%)	23 (18.7%)
Benign	5	14	19 (82.6%)
Malignant		4	4 (17.4%)
Midfoot	2 (3.8%)	51 (96.2%)	53 (43.1%)
Benign	2	37	39 (73.6%)
Malignant	0	14	14 (26.4%)
Hindfoot	3 (10.3%)	26 (89.7%)	29 (23.6%)
Benign	3	24	27 (93.1%)
Malignant		2	2 (6.9%)
Ankle	1 (5.6%)	17 (94.4%)	18 (14.6%)
Benign	1	12	13 (72.2%)
Malignant		5	5 (27.8%)
Overall	11 (8.9%)	112 (91.1%)	123 (100.0%)
Benign	11 (100.0%)	87 (77.7%)	98 (79.7%)
Malignant	0 (0.0%)	25 (22.3%)	25 (20.3%)

## Data Availability

The datasets used and/or analysed during the current study are available from the corresponding author on reasonable request.

## References

[1] Ozdemir H.M., Yildiz Y., Yilmaz C., Saglik Y. (1997). Tumors of the foot and ankle: Analysis of 196 cases. J. Foot Ankle Surg..

[2] Rammelt S., Fritzsche H., Hofbauer C., Schaser K.D. (2020). Malignant tumours of the foot and ankle. Foot Ankle Surg..

[3] Van Hul E., Vanhoenacker F., Van Dyck P., De Schepper A., Parizel P.M. (2011). Pseudotumoural soft tissue lesions of the foot and ankle: A pictorial review. Insights Imaging.

[4] Toepfer A., Lenze U., Holzapfel B.M., Rechl H., von Eisenhart-Rothe R., Gollwitzer H. (2012). Tumors of the foot: Diagnostics and therapy. Orthopade.

[5] Brotzmann M., Hefti F., Baumhoer D., Krieg A.H. (2013). Do malignant bone tumors of the foot have a different biological behavior than sarcomas at other skeletal sites?. Sarcoma.

[6] Young P.S., Bell S.W., MacDuff E.M., Mahendra A. (2013). Primary osseous tumors of the hindfoot: Why the delay in diagnosis and should we be concerned?. Clin. Orthop. Relat. Res..

[7] Ozer D., Aycan O.E., Er S.T., Tanritanir R., Arikan Y., Kabukcuoglu Y.S. (2017). Primary Tumor and Tumor-Like Lesions of Bones of the Foot: Single-Center Experience of 166 Cases. J. Foot Ankle Surg..

[8] Karaca M.O., Basarir K., Merter A., Acar E., Ozbek E.A., Ozyildiran M., Yildiz H.Y. (2022). Malignant Tumors of the Foot and Ankle. Foot Ankle Int..

[9] Angelini A., Biz C., Cerchiaro M., Longhi V., Ruggieri P. (2023). Malignant Bone and Soft Tissue Lesions of the Foot. J. Clin. Med..

[10] Toepfer A., Harrasser N., Recker M., Lenze U., Pohlig F., Gerdesmeyer L., von Eisenhart-Rothe R. (2018). Distribution patterns of foot and ankle tumors: A university tumor institute experience. BMC Cancer.

[11] Clauser C., McConville J., Young J. (1969). Weight, Volume, and Center of Mass of Segments of the Human Body.

[12] Plagenhoef S. (1983). Anatomical data for analyzing human motion. Res. Q Exerc. Sport..

[13] Ruggieri P., Angelini A., Jorge F.D., Maraldi M., Giannini S. (2014). Review of foot tumors seen in a university tumor institute. J. Foot Ankle Surg..

[14] Chou L.B., Ho Y.Y., Malawer M.M. (2009). Tumors of the foot and ankle: Experience with 153 cases. Foot Ankle Int..

[15] Biscaglia R., Gasbarrini A., Bohling T., Bacchini P., Bertoni F., Picci P. (1998). Osteosarcoma of the bones of the foot—An easily misdiagnosed malignant tumor. Mayo Clin. Proc..

[16] Kransdorf M.J. (1995). Malignant soft-tissue tumors in a large referral population: Distribution of diagnoses by age, sex, and location. AJR Am. J. Roentgenol..

[17] Pollandt K., Werner M., Delling G. (2003). Tumors of the footbones—A report from the Hamburg Bone Tumor Registry. Z. Orthop. Ihre Grenzgeb..

[18] Kransdorf M.J. (1995). Benign soft-tissue tumors in a large referral population: Distribution of specific diagnoses by age, sex, and location. AJR Am. J. Roentgenol..

[19] Karadeniz S., Yurtbay A., Albayrak B., Buyukceran I., Dabak N. (2022). A Study to Determine the Incidence and Distribution Patterns of Foot and Ankle Tumors in Bone and Soft Tissue. Cureus.

[20] Buchner M., Bernd L., Zahlten-Hinguranage A., Sabo D. (2005). Bone and soft-tissue tumors of the foot and ankle. Chirurg.

[21] Singer A.D., Datir A., Tresley J., Langley T., Clifford P.D., Jose J., Subhawong T.K. (2016). Benign and malignant tumors of the foot and ankle. Skeletal Radiol..

[22] Khan Z., Hussain S., Carter S.R. (2015). Tumours of the foot and ankle. Foot.

[23] Healey J.H., Turnbull A.D., Miedema B., Lane J.M. (1986). Acrometastases. A study of twenty-nine patients with osseous involvement of the hands and feet. J. Bone Jt. Surg. Am..

[24] Greco T., Cianni L., De Mauro D., Dughiero G., Bocchi M.B., Cazzato G., Ragonesi G., Liuzza F., Maccauro G., Perisano C. (2020). Foot metastasis: Current knowledge. Orthop. Rev..

[25] Toepfer A.K.S., Meester J. (2018). Unicameral bone cyst of the calcaneus. OUP.

[26] Tins B.J., Berkowitz Y.J., Konala P., Davies M., Cassar-Pullicino V.N., Lalam R., Cool P. (2021). Intraosseous lipomas originating from simple bone cysts. Skeletal Radiol..

[27] Malghem J., Lecouvet F., Vande Berg B. (2017). Calcaneal cysts and lipomas: A common pathogenesis?. Skeletal Radiol..

[28] Toepfer A., Strassle M., Lenze U., Lenze F., Harrasser N. (2023). Allogenic Cancellous Bone versus Injectable Bone Substitute for Endoscopic Treatment of Simple Bone Cyst and Intraosseous Lipoma of the Calcaneus and Is Intraosseous Lipoma a Developmental Stage of a Simple Bone Cyst?. J. Clin. Med..

[29] Fechner R.M.S.E. (1993). Tumors of the bones and joints. Atlas of Tumor Pathology, Fascicle 8, Third Series.

[30] Dahlin D.C., Unni K.K. (1996). Dahlin’s Bone Tumors: General Aspects and Data on 11,087 Cases.

[31] Huvos A. (1991). Bone Tumors: Diagnosis, Treatment, and Prognosis.

[32] Chou L.B., Malawer M.M. (1994). Analysis of surgical treatment of 33 foot and ankle tumors. Foot Ankle Int..

[33] Cawte T.G., Steiner G.C., Beltran J., Dorfman H.D. (1998). Chondrosarcoma of the short tubular bones of the hands and feet. Skeletal Radiol..

[34] Dahlin D.C., Salvador A.H. (1974). Chondrosarcomas of bones of the hands and feet--a study of 30 cases. Cancer.

[35] Murari T.M., Callaghan J.J., Berrey B.H., Sweet D.E. (1989). Primary benign and malignant osseous neoplasms of the foot. Foot Ankle.

[36] Pachter M.R., Alpert M. (1964). Chondrosarcoma of the Foot Skeleton. J. Bone Jt. Surg. Am..

[37] Wiss D.A. (1983). Chondrosarcoma of the first metatarsal. J. Surg. Oncol..

[38] Gajewski D.A., Burnette J.B., Murphey M.D., Temple H.T. (2006). Differentiating clinical and radiographic features of enchondroma and secondary chondrosarcoma in the foot. Foot Ankle Int..

[39] Coughlin M.J., Saltzman C.L., Mann R.A. (2013). Soft tissue and bone tumors. Mann’s Surgery of the Foot & Ankle.

[40] Evans S., Ramasamy A., Jeys L., Grimer R. (2014). Delayed diagnosis in metastatic lesions of the foot. Ann. R Coll. Surg. Engl..

[41] Choong P.F., Qureshi A.A., Sim F.H., Unni K.K. (1999). Osteosarcoma of the foot: A review of 52 patients at the Mayo Clinic. Acta Orthop. Scand..

[42] Toepfer A. (2017). Tumors of the foot and ankle—A review of the principles of diagnostics and treatment. Fuß Sprunggelenk.

[43] Adkins C.D., Kitaoka H.B., Seidl R.K., Pritchard D.J. (1997). Ewing’s sarcoma of the foot. Clin. Orthop. Relat. Res..

[44] Mendenhall W.M., Mendenhall C.M., Reith J.D., Scarborough M.T., Gibbs C.P., Mendenhall N.P. (2006). Pigmented villonodular synovitis. Am. J. Clin. Oncol..

[45] Mastboom M.J.L., Palmerini E., Verspoor F.G.M., Rueten-Budde A.J., Stacchiotti S., Staals E.L., Schaap G.R., Jutte P.C., Aston W., Gelderblom H. (2019). Surgical outcomes of patients with diffuse-type tenosynovial giant-cell tumours: An international, retrospective, cohort study. Lancet Oncol..

[46] Fraser E.J., Sullivan M., Maclean F., Nesbitt A. (2017). Tenosynovial Giant-Cell Tumors of the Foot and Ankle: A Critical Analysis Review. JBJS Rev..

[47] Noailles T., Brulefert K., Briand S., Longis P.M., Andrieu K., Chalopin A., Gouin F. (2017). Giant cell tumor of tendon sheath: Open surgery or arthroscopic synovectomy? A systematic review of the literature. Orthop. Traumatol. Surg. Res..

[48] Siegel M., Bode L., Sudkamp N., Kuhle J., Zwingmann J., Schmal H., Herget G.W. (2021). Treatment, recurrence rates and follow-up of Tenosynovial Giant Cell Tumor (TGCT) of the foot and ankle-A systematic review and meta-analysis. PLoS ONE.

[49] Johnson E.W., Ghormley R.K., Dockerty M.B. (1956). Hemangiomas of the extremities. Surg. Gynecol. Obstet..

[50] Pozarny E., Grandi R., Lane G. (2001). Venous aneurysm of the dorsal venous arch. J. Am. Podiatr. Med. Assoc..

[51] Mascard E., Gaspar N., Brugieres L., Glorion C., Pannier S., Gomez-Brouchet A. (2017). Malignant tumours of the foot and ankle. EFORT Open Rev..

[52] Murphey M.D., Ruble C.M., Tyszko S.M., Zbojniewicz A.M., Potter B.K., Miettinen M. (2009). From the archives of the AFIP: Musculoskeletal fibromatoses: Radiologic-pathologic correlation. Radiographics.

[53] Weiss S., Goldblum J., Weiss S.W., Goldblum J.R. (2001). Fibromatoses. Enzinger and Weiss’s Soft-Tissue Tumors.

[54] Hao X., Levine D., Yim J., Qi C., Firestone L., Beiser I., Leone E., Woelffer K., Mirkin G. (2019). Schwannoma of Foot and Ankle: Seven Case Reports and Literature Review. Anticancer. Res..

[55] Carvajal J.A., Cuartas E., Qadir R., Levi A.D., Temple H.T. (2011). Peripheral nerve sheath tumors of the foot and ankle. Foot Ankle Int..

[56] Azevedo C.P., Casanova J.M., Guerra M.G., Santos A.L., Portela M.I., Tavares P.F. (2013). Tumors of the foot and ankle: A single-institution experience. J. Foot Ankle Surg..

[57] Kehoe N.J., Reid R.P., Semple J.C. (1995). Solitary benign peripheral-nerve tumours. Review of 32 years’ experience. J. Bone Jt. Surg. Br..

[58] Kirby E.J., Shereff M.J., Lewis M.M. (1989). Soft-tissue tumors and tumor-like lesions of the foot. An analysis of eighty-three cases. J. Bone Jt. Surg. Am..

[59] Bermejo A., De Bustamante T.D., Martinez A., Carrera R., Zabia E., Manjon P. (2013). MR imaging in the evaluation of cystic-appearing soft-tissue masses of the extremities. Radiographics.

[60] Friedman M.V., Kyriakos M., Matava M.J., McDonald D.J., Jennings J.W., Wessell D.E. (2013). Intra-articular synovial sarcoma. Skeletal Radiol..

[61] Walker E.A., Song A.J., Murphey M.D. (2010). Magnetic resonance imaging of soft-tissue masses. Semin. Roentgenol..

[62] Durbec F., Martin L., Derancourt C., Grange F. (2012). Melanoma of the hand and foot: Epidemiological, prognostic and genetic features. A systematic review. Br. J. Dermatol..

[63] Adams B.E., Peng P.D., Williams M.L. (2018). Melanoma of the Foot Is Associated With Advanced Disease and Poorer Survival. J. Foot Ankle Surg..

[64] Toepfer A., Lenze U., Harrasser N. (2016). Calcaneal Ossoscopy. Arthrosc. Tech..

[65] Pogoda P., Priemel M., Linhart W., Stork A., Adam G., Windolf J., Rueger J.M., Amling M. (2004). Clinical relevance of calcaneal bone cysts: A study of 50 cysts in 47 patients. Clin. Orthop. Relat. Res..

[66] Macdonald D.J., Holt G., Vass K., Marsh A., Kumar C.S. (2007). The differential diagnosis of foot lumps: 101 cases treated surgically in North Glasgow over 4 years. Ann. R Coll. Surg. Engl..

